# Climate change adapted rice production: does the system of rice intensification impact malaria vector ecology?

**DOI:** 10.1186/s13071-025-06973-y

**Published:** 2025-10-24

**Authors:** Harrison Hardy, Richard J. Hopkins, Ladslaus Mnyone, Frances M. Hawkes

**Affiliations:** 1 Natural Resources Institute, University of Greenwich, Chatham, UK; 2 Institute of Pest Management, Sokoine University of Agriculture, Morogoro, Tanzania; 3 Department of Science, Technology and Innovation, Ministry of Education, Science and Technology, Dar es Salaam, Tanzania

**Keywords:** System of rice intensification, Malaria, *Anopheles*, Climate change

## Abstract

The proliferation of malaria vectors from irrigated rice crop systems has long been known, though the relationship between rice cultivation and malaria transmission is historically complex. Despite this, contemporary research reveals an association between enhanced malaria vector densities, originating from rice fields, and intensified malaria transmission in rice-associated communities is now occurring. In the wake of the ever-increasing pressures of anthropogenic climate change and a desire to increase rice production across the continent of Africa, alternative rice cultivation practices are being employed. One such alternative practice is the System of Rice Intensification (SRI), which although agronomically contentious is utilised in an attempt to enhance rice yields whilst reducing agricultural inputs, including water. SRI fundamentally alters the rice growing environment and may therefore have significant impacts on the ecology of malaria vector species. As a result, there may be important consequences for local malaria transmission dynamics. The adoption of SRI across Africa is increasing and is likely to do so further in the wake of the pressures of climate change. In this review, we critically discuss the possible impacts of SRI practice on the bionomics of the dominant malaria vector species of Africa.

**Graphical Abstract:**

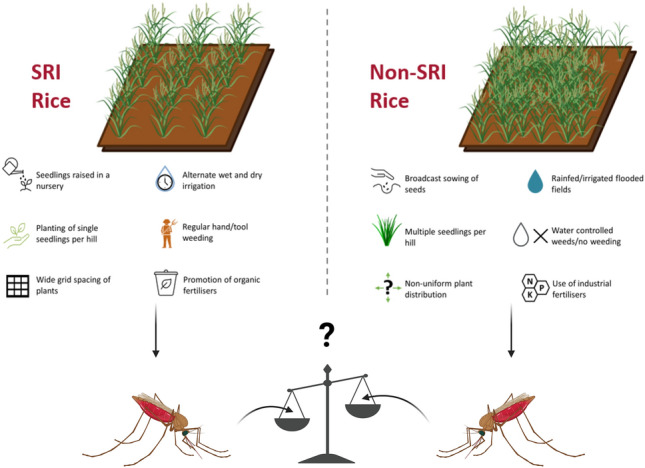

Created in BioRender. Hardy, H. (2025) https://BioRender.com/mo3g1wr

## Background

The hematophagous behaviour of female mosquitoes presupposes their susceptibility to acquiring pathogens and passing them on to humans, such as in the case of *Anopheles* mosquitoes and malaria parasites. Despite considerable reductions in malaria cases in the past two decades, malaria continues to cause the highest death toll of any vector-borne disease globally. Africa has historically and presently endured the majority of this burden, and children under five and pregnant women are at the highest risk. Almost half of the global population, across 83 countries, was at risk of malaria in 2023, and an estimated 263 million cases were reported worldwide [1]. Of the 597,000 estimated deaths due to malaria in 2023, the African region accounted for over 95% and, in this region, children under five accounted for 76% of all deaths [1]. Although considerable progress in the control of malaria in the past century has been made, in recent years the effect of such efforts has plateaued and even worsened in the years immediately after the COVID-19 pandemic compared to those before [1].

For a mosquito to be an efficient vector, it must be closely associated with its host, and its longevity sufficient to allow the pathogen or parasite to produce infective stages in adequate quantities for transmission [2]. However, the overall system of malaria transmission is a product of and subject to continuous change through the evolution and development of its components: *Anopheles* mosquitoes, humans, *Plasmodium* parasites*,* and the changing environment.

While suitable habitats for malaria vectors are abundant in nature, human alterations to the landscape can unintentionally create ideal breeding grounds for anopheline mosquitoes. In particular, the proliferation of *Anopheles* species and rice cultivation are highly associated [3]. The flooding of rice fields provides conducive aquatic habitats that the larvae of *Anopheles gambiae* sensu lato (s.l.), specifically *An. gambiae* s.s., *An. arabiensis,* and *An. coluzzii*, are well-adapted to exploit [4]. Moreover, irrigation of rice may extend the breeding season of mosquitoes beyond their normal timeframe, depending on local seasonal climatic conditions and the number of cropping cycles [5]. Rice cultivation, therefore, can provide large areas of suitable breeding habitat, leading to higher densities of malaria vectors than would otherwise be the case [6]. However, the relationship between rice cultivation, particularly irrigated rice, and malaria is complex, and increased vector densities have not historically been associated with enhanced malaria transmission in all cases [5], although recent analyses have revealed that communities associated with rice irrigation do indeed experience a higher malaria burden [7]. Despite this, differences in rice cultivation methods, such as flooded and non-flooded irrigation, are associated with significant differences in malaria incidence in associated communities [6], demonstrating a clear need to elucidate how varying cultivation practices may impact this dynamic.

With a rapidly growing population, many countries in sub-Saharan Africa have increased the production of rice and between 2008 and 2018; the Coalition for African Rice Development (CARD) policy framework aimed to double rice production in sub-Saharan Africa. The region ultimately achieved a 103% increase in this timeframe and is forecast to continue to rise into 2030 with the goal of self-sufficiency. This increase in production has been attributed largely to enhanced yields rather than the expansion of the total rice cultivation area [8]. Among many other methodologies, the System of Rice Intensification (SRI) is a suite of integrated practices developed to increase rice yields whilst reducing agricultural inputs and, ultimately, altering the rice-growing agroecosystem [9]. Some authors report yield increases from 25 to above 100%, depending on the rice cultivar, with the utilisation of 25–50% less water [10–12], though these claims are not without controversy [13–15]. Nevertheless, SRI continues to be promoted and adopted by resource-poor farmers as a means of increasing yields whilst reducing costs [16], and more generally as a climate change-adapted rice cultivation technique [17, 18].

Through intended environmental modifications, SRI practice may also fundamentally alter the mosquito larval habitat and may, therefore, impact the ecology and biology of malaria vector populations breeding in rice fields. Whilst the impact of SRI on crop pests and insect biodiversity has been addressed [18–20], there has been very little research focusing on how the practice holistically may affect *Anopheles* mosquitoes, as no peer-reviewed publications could be identified that focus on the subject. However, a single Master’s thesis reported high larval mortalities in SRI fields, relative to conventional rice crop systems [21]. Still, some of the components of SRI have garnered some research attention, particularly alternate wet and dry (AWD) irrigation, which has been demonstrated to reduce *Anopheles* larval densities [22] and has been suggested as a potential vector control strategy [23, 24]. However, in some cases, farmers have resisted adopting AWD due to concerns that it will increase rodent pest activity, particularly that which results in rice yield losses, despite recent evidence that the practice has no impact on rodent-induced crop damage [25].

With the purported benefits to crop yield with fewer agricultural inputs, reduced water utilisation, and the potential to mitigate greenhouse gas emissions associated with SRI, its adoption will likely continue to rise in the face of the ever-increasing twin challenges presented by anthropogenic climate change and food security [9, 26, 27]. Despite this, the potential for SRI to modify the ecology and biology of *Anopheles* mosquitoes, and therefore their ability to transmit malaria, remains uncertain. Given the close association between the rice agroecosystem and *Anopheles* mosquitoes [7], and the dependence of malaria transmission on vector physiology and ecology [28], it is important to study these interrelationships in the context of vector control. Whether SRI leads to the repression or promotion of vector populations and the resultant reduction or exacerbation of malaria transmission, respectively, demands urgent attention.

This review will outline the ecology of the dominant malaria vector species in Africa, within the context of rice cultivation and malaria transmission, give a brief introduction to the broad concepts of SRI, and discuss the potential impacts of SRI on the ecology of malaria vectors.

### Dominant vector species of Africa and their larval ecology

Undoubtedly, our advances in the control of *Anopheles* mosquitoes and malaria manifested from our knowledge of vector species biology and ecology. Understanding how vector mosquitoes behave, where they occur, and how they interact with their environment has allowed us to reduce the transmission of malaria by exploiting key components of their ecology and biology.

Members of the *An. gambiae* species complex comprise some of the world’s most efficient vectors of malaria. In sub-Saharan Africa, *An. gambiae* s.s., *An. arabiensis*, and *An. funestus* s.l. are considered the dominant vector species (DVS) of the region [29], however, their distribution and relative importance to malaria transmission differ based on local geography and climate. *An. gambiae* s.s. is prevalent in humid forested zones, whereas *An. arabiensis* occurs in drier savannah zones due to their adaptation to this climatic niche [30, 31]. Although *An. gambiae* s.s. and *An. arabiensis* are more dominant than *An. funestus* s.l. overall, in some regions the latter species accounts for nearly 90% of all infective bites at relatively lower population densities, suggesting a relatively greater vectorial capacity [32].

Historically, *An. gambiae* s.s. has been described as the main vector species of Sub-Saharan Africa, owing to its high anthropophily and, hence, vectorial capacity [28, 33]. However, with the mass distribution of LLINs and the use of IRS, marked declines in vector densities have seen a disproportionate reduction in *An. gambiae* s.s. in comparison to *An. arabiensis*, leading to a shift in species composition and predominance of *An. arabiensis* in some regions [34]. The insecticides used to treat LLINs appear to be less effective at killing *An. arabiensis* than both *An. gambiae* s.s. and *An. funestus* s.l. [35, 36], likely due to higher behavioural plasticity and insecticide resistance [37]. Nevertheless, *An. gambiae* s.s. maintains its status as a DVS, and regional shifts in species composition are likely to continue as insecticide resistance proliferates [38, 39].

**Fig. 1:**
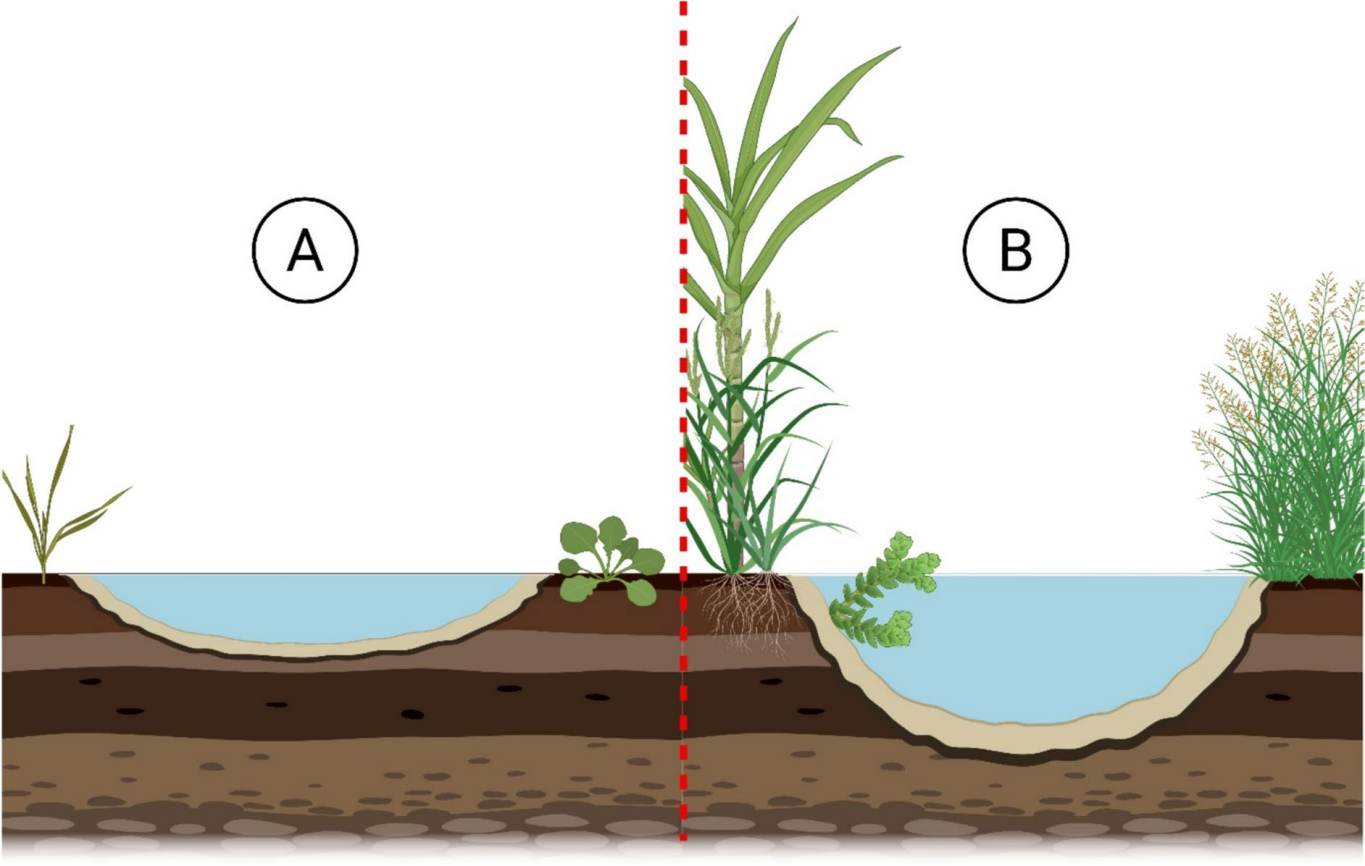
Diagram representing the basic characteristics of *An. gambiae s.s.* and *An. arabiensis* (**A**), and *An. funestus s.l.* (**B**) larval habitats. Created in BioRender. Hardy, H. (2025) https://BioRender.com/g21k058

The availability and suitability of larval habitats are key determinants of adult mosquito density, distribution, and fitness [40–44] and, consequently, malaria transmission [28, 45]. Hence, before the discovery and mass use of Dichlorodiphenyltrichloroethane (DDT), larval source management was the predominant method of malaria vector control [46]. The larval habitats of *An. arabiensis* and *An. gambiae* s.s. are typically shallow, fresh, unpolluted, sunlit water bodies that are small in size with little vegetation [47] (Fig. 1). These breeding sites are usually ephemeral, ranging in size from foot or hoofprints to drainage ditches, cultivated swampland, and irrigated crop systems such as rice fields [48–50]. *An. funestsus* s.l. is described as breeding in semi-permanent and permanent bodies of water that are shaded by abundant vegetation [51], such as ponds, river edges, savannah, sugar cane plantations, and late-growth rice fields [5, 6, 52].

### Rice cultivation, *Anopheles* mosquitoes, and malaria

Rice cultivation has long been associated with malaria as the rice agroecosystem provides suitable larval habitats whilst human settlements are often nearby, providing a bloodmeal source for adults [3, 53]. Moreover, rice-associated volatile semiochemicals attract gravid female *Anopheles* mosquitoes and stimulate them to oviposit [54]. All three of the African DVS can be found inhabiting the rice agroecosystem, among other Culicidae, where a succession of species may be observed in areas in which the three are sympatric. Both *An. arabiensis* and *An. gambiae* s.s. may be present in rice fields during the field preparation phase, with their densities typically peaking during the first six weeks of growth post-transplantation. Thereafter, as rice height continues to increase, canopy closure of the vegetation occurs (around weeks 6–8), creating a more shaded environment, in which time *An. gambiae* s.l. may be completely absent and *An. funestus* s.l. predominant [55, 56]. After rice harvest, improper field levelling and drainage may result in the formation of shallow sunlit pools, which *An. gambiae* s.l. readily colonise [57].

In comparison to other common agroecosystems associated with *Anopheles* mosquitoes, rice cultivation has been found to support the highest abundance of malaria vector species, although the relative abundance can depend on water management practices. In Tanzania, Mboera *et al*. [6] captured significantly higher numbers (70.7%) of adult *An. gambiae s.l.* and *An. funestus* s.l. over eleven months in a flooded field rice agroecosystem, compared to irrigated non-flooding rice (8.6%), sugarcane (7.0%), wet savannah (7.3%), and dry savannah (6.4%) agroecosystems. Similarly, in Kenya, Mwangangi *et al*. [49] found relatively lower abundances of anopheline larvae in sample sites located in irrigated rice agroecosystems in comparison to those that were primarily flooded fields, rainfed, or supplementarily irrigated by local streams. Consequently, rice agroecosystems support higher biting rates, with up to 46 bites per person per night recorded in communities associated with irrigated rice cultivation in some instances, compared to less than five bites per person per night in association with farmed savannah [58].

Malaria incidence is over six times higher in irrigated rice cultivation communities compared to those associated with pastoral savannah [58]. Similar trends were observed by Rumisha *et al.* [59], whose cross-sectional malaria screening study in the Mvomero district of Tanzania found 78.1% of those infected were from communities associated with irrigated rice cultivation, 18.7% from Savannah, and only 3.2% from sugarcane agroecosystems. Given the findings of Rumisha *et al.* [59], it seems clear that of the common agroecosystems associated with *Anopheles* species in Africa, rice cultivation supports the largest vector populations, human biting rates, and malaria risk, though flooded field systems may pose a relatively greater risk than irrigated systems.

The development of rice irrigation schemes and the risk of malaria transmission is, however, complex and debated in the associated literature. Irrigation channels and the rice fields they supply provide conducive anopheline larval habitats, can prolong breeding seasons, and ultimately lead to the development of increased population densities [60]. An increased population density, holding all other factors constant, would lead to a higher vectorial capacity for a given population [28], and therefore rice irrigation-associated communities would be expected to bear a higher burden of malaria. However, in their landmark study, Ijumba and Lindsay [5] found this is not always the case and communities located close to irrigated rice cultivation may even experience a reduced malaria case incidence compared to those in surrounding areas, despite simultaneously experiencing elevated biting rates. This unexpected phenomenon was termed the “paddies paradox” and several possible explanations may be responsible.

Bed net usage increases in irrigated rice cultivation-associated communities in response to the larger numbers of mosquitoes entering households at night [61], which effectively reduces the biting rate during the hours of sleep and consequently the malaria incidence rate [62]. Moreover, blood feeding is density-dependent, with individuals finding difficulty in attaining a blood meal at high population densities, particularly when bed nets are used, eliciting behavioural changes in feeding such as increased zoophagy [63], thus leading to reduced transmission. Further, Ijumba and Lindsay [5] also posited that in some cases the more competent vector, *An. funestus* s.l.*,* may be locally displaced by *An. arabiensis* as the latter species is more suited to the rice field habitat [49]. Lastly, and most prominently, the development of irrigation systems for rice cultivation is associated with socioeconomic benefits in associated communities, where enhanced wealth leads to improved bed net and healthcare access, resulting in reduced malaria case incidence [5]. Though in some cases Ijumba and Lindsay [5] found increased malaria transmission in rice-growing communities, these were attributed to areas of unstable or seasonal transmission, where the human population had little immunity and in some cases had transformed into a stable or perennial transmission system following irrigation development [64, 65].

Since the publication of the “paddies paradox”, considerable reductions in malaria incidence have occurred across Africa with the widespread coverage of modern control strategies [66, 67]. Despite this, we have recently observed a slowdown in malaria case reduction, believed to be brought about by previously effective control measures having reduced efficacy due to behavioural changes and insecticide resistance in vector species [35, 37, 68]. Studies in the past two decades have consistently reported increased malaria transmission risk in association with rice cultivation in areas of stable transmission [6, 58, 59, 69]. Subsequently, a recent meta-analysis of observational studies conducted across 14 different African countries in Sub-Saharan Africa revealed that whilst malaria prevalence was not higher in irrigated rice communities in studies performed before 2003, post-2003 malaria prevalence was 70% higher than in surrounding communities [7]. This may be attributed to the mass rollout of LLINs across sub-Saharan Africa [66], leading to comparable vector protection across communities, revealing the extent to which greater vector proliferation due to rice agriculture enhanced malaria transmission. This demonstrates that irrigated rice cultivation now, contrary to the conclusions of the “paddies paradox”, may increase the risk of malaria in associated communities.

With growing rice production and increased reliance on irrigation, further research is urgently required to improve rice cultivation practices without increasing the malaria burden, especially for new, climate-adapted techniques like SRI, which are being adopted and actively promoted without an understanding of their effect on anopheline ecology and, consequently, malaria transmission.

### The system of rice intensification

SRI was developed as a means to increase rice yields in Madagascar by Henri de Laulanié [70] as a response to the large investments, expensive equipment, and infrastructure required for irrigated rice production, which are commonly inaccessible to smallholder farmers across Africa [9]. SRI can also provide improved yields over conventional irrigated or rain-fed cultivation, whilst also reducing water usage and other agricultural inputs [71]. With the mounting pressures of increasing populations, impacts of climate change, and reducing landholdings, a resurgence of interest in low external input intensification practices, such as SRI, is occurring in sub-Saharan Africa [72]. SRI is not considered a single technology, but a “set of interdependent agronomic practices that modify current plant, soil, water, and nutrient management” [26] and it is through this that SRI cultivation systems diverge fundamentally from more conventional irrigated systems and flooded field cultivation systems.

**Fig. 2:**
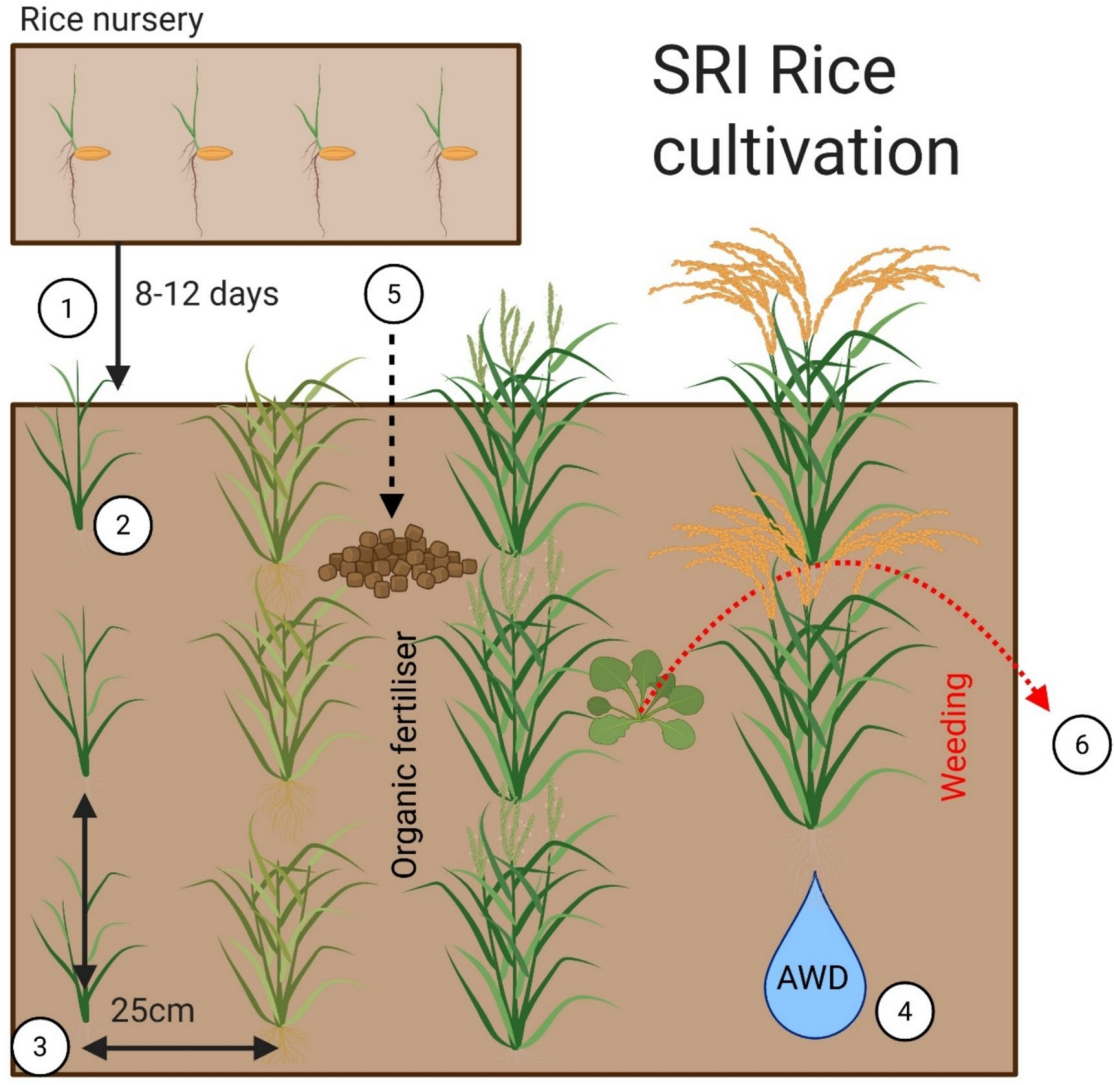
Diagram of the System of Rice Intensification (SRI) integrated cultivation practices. Numbers in white circles correspond with field practices used in SRI rice cultivation: (1) transplant of young seedlings; (2) transplant of a single rice plant per hill; (3) wide grid-spacing of individual rice plants; (4) alternate wetting and drying regimes; (5) addition of organic fertilisers; (6) early and regular weeding. Created in BioRender. Hardy, H. (2025) https://BioRender.com/a69x034

SRI practice consists of three main principles which are applied to field practice, covering the entirety of the rice growth period: (1) planting younger seedlings, (2) planting seedlings at optimal density, and (3) maintaining soils in mostly aerobic conditions [26]. Most publications describe SRI practice as having six core practices [9, 26, 73], although others may combine some principles and reduce the number [18], while some extend these to as many as nine [74]. Regardless, the core ideas of SRI (Fig. 2) remain consistent and can be summarised in terms of six field practices:

1. Transplant of young seedlings (8–12 days) from a nursery, carefully and quickly (no more than 15–30 min from nursery to field), and at a shallow depth of 1–2 cm.
2. Transplant of a single rice plant per planting hill, instead of multiple plants.
3. The wide spacing of individual rice plants (~ 25 cm apart) in a grid fashion.
4. Carefully controlled water management to create mostly aerobic soil conditions through alternate wetting and drying regimes (AWD).
5. Addition of organic fertilisers (as opposed to industrially produced fertilisers) such as compost or manure to enhance soil fertility.
6. Early and regular weeding, either by hand or mechanical weeding tools.

SRI farming is also divergent from conventional rice cultivation in that it does not typically employ the use of chemical pesticides, herbicides, or fertilisers [74]. However, it has been suggested that SRI would benefit from the addition of chemical fertilisers [75], and with many rice production systems utilising fertilisers already in sub-Saharan Africa [72] it would not be unreasonable to suggest that SRI and chemical fertiliser application are already, or will be, employed together in some cases.

### The potential impact of SRI on *Anopheles* ecology

There has been little published research on the impact of SRI on mosquito ecology and to date, only a single Master’s thesis has formally addressed the subject [21]. However, some of the individual practices of SRI, such as AWD, have been studied somewhat, but the impact of the individual components largely remains unclear. Furthermore, when combined and integrated into SRI cultivation, each of its practices may interact with one another to modify the environment and, therefore, the habitat of juvenile mosquito stages in ways they may not have individually. The consequences for regional malaria burden due to changes in vector ecology demand attention due to SRI’s increasing popularity in sub-Saharan Africa.

#### Light intensity

One possible outcome of SRI on the environmental characteristics of the rice field is a reduction in the amount of shade within the cropped area. Conventional rice cropping utilises either broadcast sowing, whereby rice seeds are semi-randomly scattered by hand, or the field is populated with rice plants raised in a nursery that are transplanted three or four seedlings to a mound [73]. In contrast, SRI promotes a reduced plant density, whereby single seedlings are transplanted per mound and widely spaced in a grid pattern. SRI fields typically support 16–25 plants/m^2^, whereas conventional cropping supports up to 150 plants/m^2^ [9]. SRI’s lower plant density may therefore allow a greater intensity of light to reach the soil and water as reduced rice plant density can increase light penetration [76]. Should wider plant spacing lead to increased light penetration SRI fields may be more attractive to gravid *An. gambiae* s.l. than *An. funestus* s.l., and the former may, therefore, predominate over the entirety of the rice growth period.

A study in The Gambia found when vegetation shaded over 25% of the aquatic habitat anopheline larval density significantly decreased by 74% [77]. This effect occurred across multiple habitat types such as rainfed flooded rice fields, man-made puddles, floodwaters and stream fringes where *An. gambiae* s.s.*, An. arabiensis* and other anopheline mosquitoes were found, indicating that decreased shading may lead to increased abundances of anopheline mosquitoes. Tuno *et al*. [78] demonstrated the greater survival of *An. gambiae* s.l. in habitats with higher light exposure, possibly due to increased production of algae, leading to reduced larval mortality [77–80]. This effect may carry over to larval habitats situated in SRI fields as the practice is posited to stimulate algal growth [81].

Mogi and Miyagi [82] found that in irrigated rice fields there was no clear association between the stage of growth, the main source of changing light intensity, and mosquito predator abundance. However, the predator abundance was correlated with larval mosquito mortality, suggesting larval abundance could be a good indicator for predation pressure, but predation pressure is not influenced by the degree of shading. Therefore, reduced shading of aquatic habitats in the SRI environment is unlikely to affect the predation of malaria vector species.

Overall, decreased shading associated with SRI practice may influence both the mosquito population and species composition. Higher light exposure may increase both larval abundance and survival through increased food provisions, leading to an increased juvenile and adult population size. Furthermore, the modified light levels found in SRI systems may favour the oviposition of *An. gambiae* s.l. over *An. funestus* s.l., an effect that may extend into the later phases of rice growth, disrupting species succession where *An. funestus* s.l. can predominate in later rice growth stages, and instead *An. gambiae* s.l. species dominating over the entire rice growth period.

#### Water temperatures

Increased light penetration resulting from reduced plant density in SRI practice may also affect the temperature of the surrounding soil substrate, and in turn, the water temperature of the larval habitat. Rice plant spacing in a 25 × 25 cm grid can lead to elevated soil surface temperatures, though these elevated temperatures may only be 1–1.5 °C greater than in higher planting densities, and this relationship is dependent on time of day [83]. Dahiru [73] asserts that AWD also leads to increased soil temperatures as the surface area of standing water is reduced, in comparison to flooded field cultivation, leading to a reduced albedo. Therefore, rice plant spacing, as promoted under SRI management, may result in elevated soil temperatures and consequently, water temperatures.

Increased temperatures reduce larval developmental periods and increase survival, though the trade-off is the production of smaller adults, which have reduced fitness [44, 84–87]. Munga *et al.* [88] compared the microclimate characteristics between three *An. gambiae s.l.* habitats and found those with the highest average water temperatures displayed the shortest larvae-to-pupae development time and the highest pupation rates. Comparatively, when studying the impacts of deforestation, Afrane *et al.* [84] found that water temperatures were 4.8–6.1 °C higher in deforested areas, due to reduced shading, and *An. gambiae* s.l. larvae experienced both shorter larval to adult development times, by 8–9 days, and increased larval survivorship of 65–82%. Furthermore, a study conducted along the Mara River, which runs through Kenya and Tanzania, found the abundance of *Anopheles* mosquitoes increased, regardless of habitat type, with increasing water temperature [89]. Lastly, larval development in elevated water temperatures has been demonstrated to enhance insecticide tolerance in both susceptible and resistant populations of *An. gambiae s.l.* [90]. These field studies indicate that should SRI increase water temperatures, it can be expected that larval survival, development, abundance, and possibly insecticide resistance, will increase.

Within the rice agroecosystem, the relationship between water temperature and *Anopheles* larval abundance seems to vary mainly with water management practices. A study which in part investigated the impact of environmental variables on rice field-inhabiting mosquitoes in Kenya found *An. pharoensis* larval abundance was positively correlated with water temperature, whereas no such correlation was observed in *An. arabiensis* [55]. In contrast, another study in Kenyan rice fields found a positive correlation between the abundance of *An. arabiensis* larvae and temperature [91]. Both of these studies were conducted in the same geographic location, the Mwea irrigation scheme, Kirinyaga district, though there was more than a year difference in the sampling period. Furthermore, both studies conducted sampling in purposefully established rice paddy sub-plots of the same spatial dimensions and hydrologically separated the subplots with unidirectional inflow and outflow gates to prevent water mixing. However, the former study stated water levels were maintained in the experimental plots at 10 cm depth throughout the sampling period (which is more reflective of a flooded field irrigation regime), whilst the latter study did not disclose any details of water management. Mwangangi *et al*. [89] did imply water depth varied as they stated that “Up to 20 dipper samples, depending on the amount of water in each subplot, were taken at intervals throughout the subplot by using a standard mosquito dipper”.

When considering water temperature, depth is an important factor as it is intimately linked with the temperature gradient in the water column and the degree to which temperature fluctuates with ambient conditions [92]. In line with this, Muturi *et al.* [55] reported mean water temperatures did not vary by more than 1.5 °C (between 27.36 and 26.25 °C) over a 14-week rice growth period, whereas Mwangangi *et al*. [91] described variations of almost 5 °C (between 29.30 and 24.56 °C) within the same period. Both studies reported the highest water temperatures in the earlier stages of the rice growth period, and the lowest in the later stages, when canopy closure would increase the shaded area, and the abundance of *An. arabiensis* larvae were highest in the early rice growth stages, reducing with increased plant growth. This suggests that the temperature range experienced by the larvae in the latter study was varied to a high enough degree that larval development and survival were significantly impacted, whereas in the former study, the temperature variation was negligible and it is likely other environmental factors played a more important role in determining larval abundance. Lyons *et al.* [86] demonstrated *An. arabiensis* survival and development rates scaled linearly with increasing temperature up to 30 °C, shedding light on the disparity between the findings of these two studies, as the larval development rate throughout the study conducted by Mwangangi *et al*. [91] would be affected by a higher degree than that of Muturi *et al.* [55]. Considering the use of AWD in SRI practice, it is likely that any standing water in such rice fields will experience a greater degree of variability in terms of its depth and temperature fluctuations, compared to a conventional flooded field system. Considering that fluctuating temperatures are detrimental to the survival and development of *An. funestus* s.s., and possibly *An. gambiae* s.s., but not *An. arabiensis*, the latter species may be more tolerant of conditions in SRI fields.

Should SRI practice lead to increased or more variable water temperatures, it seems likely that the life history traits of any *Anopheles* mosquitoes inhabiting the rice field will be affected. In general, all three DVS show increased developmental rates with increased temperatures, however, only *An. arabiensis* is unaffected by wide temperature variations and therefore the SRI-managed fields may be more conducive to the latter species. Combined with a more open canopy structure, potentially increased water temperatures, and the more transient nature of surface water under SRI management, a shift in the species composition may occur whereby *An. gambiae* s.l. predominate over the entire rice growth period and *An. funestsus s.l* abundance is reduced. Lastly, *An. arabiensis* may have a further advantage over *An. gambiae s.s.*, owing to its higher temperature threshold for survival [86], should water temperatures exceed 30 °C on average, possibly leading to *An. arabiensis* dominating the anopheline species composition.

#### The multiple impacts of alternate wet and dry irrigation (AWD)

The practice of AWD irrigation within SRI may be the most impactful determinant of mosquito ecology in comparison to conventional systems. AWD irrigation reduces the abundance of mosquitoes in some instances [24, 93] and has been suggested as a means of vector control [23]. Due to the complexities of soil drainage and climate, AWD is not always feasible as it requires rapid drying and drainage [24]. In addition, when AWD is employed, its characteristics vary greatly with some farmers adhering to a strictly regular irrigation regime [18], whilst others use visual indicators such as soil cracking to decide when to commence irrigating [72]. Though proponents of SRI claim the practice leads to greater yields, AWD alone has been found to reduce yields in some cases, or to have no effect in others [9, 17, 94]. As early as 1947, AWD was being evaluated as a means to reduce the abundance of disease vector mosquitoes [93]. However, the integration of AWD and its impact on mosquito ecology when combined with the other practices of SRI have yet to be assessed.

A study by Mutero *et al.* [94], conducted in the Mwea irrigation scheme in Kenya, highlighted the effect of AWD on the survivability of *An. arabiensis* larvae by analysing the ratio of early and late instars. The abundance of 1st instar larvae in AWD rice fields was significantly higher, in comparison to continuously flooded fields, paired with a much lower abundance of 4th instar larvae. The ratio of 4th instar to 1st instars was 0.08 in the AWD fields, whereas in the continuously flooded fields, this ranged from 0.27 to 0.68, indicating survival was between 3.3 and 8.5 times lower in the AWD plots. Still, the total number of 4th instar larvae found in AWD fields was similar to that of the conventionally irrigated fields, implying a greater number of eggs were laid in AWD fields. The inability to eliminate larval development by AWD was attributed to the formation of residual pools due to poor land management, which has also been alluded to by others [23, 57].

Contrary to these previous findings, Ijumba [57] found AWD increased mosquito production. Again, this was attributed to the formation of small pools, due to improper field levelling. Despite apparent conflicts in these findings, one factor remains consistent: poor soil drainage characteristics and improper land management can lead to the formation of small shallow pools, which can maintain high mosquito productivity regardless of AWD irrigation. Moreover, a recent study found that “fresher ponds”, aquatic habitats that are newer formed and have had less exposure to climatic effects, experience significantly higher *An. arabiensis* oviposition rates, therefore, the ongoing replenishment of water in SRI cultivation may contribute to increased larval abundances [97].

A study from southern India further exemplifies the importance and complexities associated with pool formation in AWD irrigation. Rajendran *et al.* [98] found AWD can reduce the abundance of both culicine and anopheline (*An. peditaeniatus, An. tessellatus,* and *An. barbirostris*) larvae, but the irrigation schedule is paramount in achieving this. In the study, a single AWD rice field over a two-year course was examined. In year one, re-irrigation occurred immediately when fields were dry, whereas in year two, this coincided with water availability, which occurred sporadically and often led to only partial drying. In year one, mosquito abundance compared to conventional rice cultivation was significantly lower (− 75–88%), as was mosquito predator abundance, whereas year two predator abundances were lower but mosquito abundance was higher than that of conventional cultivation, a result similar to that found by Ijumba [57]. The authors suggested this was due to an increased predator efficiency in year one caused by the crowding and localisation of their prey, whereas in year two predator abundance was decreased along with predation efficiency due to an increased mosquito habitat area facilitated by incomplete field drying [98].

Another study conducted in Japan [93], where a similar sporadic AWD schedule was applied, reflects the findings of Rajendran *et al.* [98]. Total aquatic insect abundance, including *An. sinensis*, was reduced but over time the proportion mosquitoes made up of all insects increased, indicating reduced predation pressures [93]. Conversely, another study in India found AWD irrigation led to a 1.9 times increased abundance of mosquitoes, including both *Culex* and *Anopheles* mosquitoes (*An. subpictus, An. annularis, An. vagus, An. barbirostris,* and *An. peditaeniatus*), when compared to traditional flooded field cultivation [99]. Moreover, a recent study demonstrated that *Anopheles* mosquitoes may benefit from AWD irrigation, as oviposition in newly formed pools after a dry period provides increased nutritional resources and fewer competitors and predators [100].

If pool formation leads to increased larval crowding, then the deleterious effects of inter- and intraspecific competition may reduce abundances, as both *An. gambiae* s.s. and *An. arabiensis* experience increased mortality in mixed-species assemblages in small pools [101]. However, each species responds differentially to pool size; *An. arabiensis* experiences reduced mortality in small pools vs large pools, whereas *An. gambiae* s.s. experience the opposite [101]. This implies that AWD may lead to overall reduced abundances but favour the survival of *An. arabiensis*. These studies underscore the complexity of how AWD may affect mosquito ecology, and that irrigation schedule, land management, and drainage characteristics are significant modulating factors of mosquito abundance and survival.

Nevertheless, *An. gambiae* s.l. are well adapted to ephemeral and fragmented habitats. *Anopheles gambiae* s.s. larvae can traverse up to 10 cm after hatching in moist soil to reach water, and their survival in moist soil ranges from 64 hours in first instars to 113 hours in fourth instars [102]. Furthermore, the eggs of *An. gambiae* s.l. can remain viable for up to 15 days in damp soil [52]. This illustrates the irrigation schedule for AWD schemes is significant, mosaics of small pools can support larvae that don’t hatch within, and the importance of a long enough dry period to effectively control mosquitoes. However, these factors are all modulated and limited by underlying soil hydrology characteristics.

For mosquitoes, AWD may pose three interlinked ecological challenges. The first being increased transience of the aquatic habitat—i.e., the length of time water is physically available and its replenishment frequency, which is known to modulate mosquito productivity and abundance [103]. Within SRI, AWD aims to maintain aerobic soil conditions [9] wherein soil is kept moist whilst avoiding surface water. What bodies of water that do form are often caused by poor land preparation; improper field levelling and footprints/depressions left by workers and machinery can lead to water pooling post-drainage and remain highly productive for mosquitoes [23, 57]. Though, these small ephemeral pools likely experience greater evaporation rates, due to potentially greater light exposure and surface temperatures, thus posing enhanced risks of desiccation for inhabitant mosquitoes, which is alluded to as the main driver behind reduced mosquito densities in AWD managed systems [24].

Second, whilst increased nutritional resources and fewer antagonists occurring in newly formed pools may benefit *An. gambiae s.l.* [100], increased predation efficiency [98] and competition may mitigate these potential benefits [98, 100, 101]. As the DVS of Africa have varying degrees of desiccation resistance [104], the first two issues would presumably pose a greater challenge to species with longer pre-imago developmentary periods, such as *An. funestus* s.s.*,* compared to those with shorter, such as *An. gambiae* s.l. [86]*.* The duration after the loss of surface water in rice fields before reirrigation is required depends on the groundwater table, where shallower water tables facilitate longer intervals [105]. Thus, there is a high degree of variability between rice cultivation systems employing AWD in terms of how much freshwater is available, how long it is present, and how long it is before a dry field is reirrigated, all of which are of importance to mosquito ecology. A third potential issue is reduced overall available habitat area as less water would be present in the SRI field relative to a conventional rice system, as up to 50% less water use is commonly reported [72, 74]. Reduced habitat availability may have important ramifications for the mosquito population carrying capacity, density-dependent effects, and increased predation within the limited habitat [98].

AWD by itself can significantly impact the ecology of *Anopheles* mosquitoes, however, there is no consensus among existing research on how exactly mosquitoes are affected, and even less is known about the species-specific effects. The success of AWD in reducing mosquito productivity seems to hinge strongly on the ability to control water drainage, the formation of pools after drying, and the length of time it is feasible to leave the field dry before reirrigation. AWD is capable of both increasing and decreasing the abundance of *Anopheles* mosquitoes depending on the specifics of its application and this, therefore, reflects the strong influence AWD has on the mosquito habitat in rice fields. Further still, little is known of how the combined effects of SRI practice and AWD may interact to impact mosquito ecology.

#### Water physicochemistry and microbiome

Physicochemical and microbial aspects of aquatic habitats influence the physiology and ecology of *Anopheles* species [106]. SRI is characterised by reduced agricultural inputs in comparison to conventional cultivation, typically referring to chemical fertiliser abstinence [74] and utilisation of organic fertilisers (OF) [73]. The composition of OFs varies, often comprising materials freely available to the farmers, such as manure or compost [9], and is one of the reasons SRI is promoted to resource-poor farmers [71–73]. As a result, the composition of OFs used in SRI is highly heterogeneous and poorly characterised [74]. Furthermore, it is reported that some farmers practising SRI are resistant to relying entirely on OFs, or do not have access to the required biomass recommended for soil enrichment, so may utilise chemical fertilisers wholly or in conjunction with OFs [73].

In addition to the use of OFs, the water quality in SRI cultivation may be influenced by weeding, which can aerate the soil and potentially the water too, whilst AWD additionally aims to maintain aerobic conditions [9]. Increased rice tiller development observed in SRI, which is linked to reduced root hypoxia [9], provides evidence for this. Additionally, SRI reduces populations of methanogenic bacteria, which require anaerobic conditions, whilst also promoting methanotrophic bacteria, which require aerobic conditions [107]. Elevated NO_3_^-^ concentrations are reported in SRI compared to conventional cultivation, where NH_4_^+^ predominates from chemical fertiliser application and a lack of oxidative conversion [108].

The addition of OFs will significantly alter the organic material content of the aquatic habitat and underlying soil. Most *Anopheles* species are known to avoid ovipositing in water contaminated with organic material such as dung or rotting vegetation [109], however, a growing body of evidence indicates not only can populations of *Anopheles* species breed in organically polluted aquatic habitats [110–112], they may also experience increased development rates and fitness in such environments [113]. However, in the case of manure-based organic fertilisers, the relative benefits of such seem highly dependent on the type of manure. Recent research demonstrates the presence of cow dung enhances larval performance of malaria vector species, both *An. arabiensis* and *An. gambiae s.s.* develop into larger adults whilst experiencing no effect on their survival and adult production, whilst the latter species may also develop at a faster rate. However, exposure to chicken dung in these species induces significant mortality, subsequent reductions in adult production, and reduces development rate [114]. Interestingly, whilst chicken dung induces this negative effect, surviving adults are larger, as with cow dung, suggesting both may act as additional nutritional resources [44, 115]. Despite this, *An. arabiensis* are less likely to oviposit in such sites in favour of those devoid of cow dung. This was also found chicken dung, though, when limited to sites containing only cow or chicken dung, greater oviposition rates occur in the former [116], suggesting the latter is relatively more deterrent to gravid females, reflecting the underlying toxicity of chicken manure and conforming to the larval-preference performance hypothesis [117, 118].

Similarly, vegetative material as OFs can have deleterious effects. A Kenyan study found reduced abundances of *An. gambiae* s.l. larvae in natural wetland and forest habitats compared to farmlands [119]. Decomposing organic matter, such as leaf litter, was implicated as it produces toxic breakdown products, such as polyphenols and other secondary compounds, that are harmful to some mosquito species, such as *An. stephensi*, and may repel gravid females [120, 121]. Application of cut grass has been utilised in India for the control of *Anopheles* mosquitoes, though found ineffective for Afrotropical species. Success in India was found in conventional flooded fields, where anaerobic bacteria break down the vegetative material, and may not apply to SRI [109]. Munga *et. al.* [119] also stress the importance of microbial communities, facilitated by decaying organic matter, that can elicit both attractive and repulsive behavioural responses in *Anopheles* species [122, 123]. Despite the importance of microbially-mediated oviposition behaviour, the only microbially excreted compound yet to be identified is cedrol [124], an oviposition attractant of African *Anopheles* species produced by two fungi species associated with the rhizomes of *Cyprus rotundus.* Although not a product of organic material decomposition, this is a clear indication of the complex and important role microbial communities play and that similar compounds associated with decomposing OFs used in SRI may also impact oviposition behaviour. Furthermore, should undiscovered microbial communities exist in the rice rhizosphere, SRI may impact them, and any influence on mosquitoes, via improved oxygen availability and greater root development [9].

Despite the aim of mostly aerobic conditions in SRI, the decomposition of organic materials in aquatic habitats causes dissolved oxygen depletion locally, via microbial metabolism. Under SRI conditions, the addition of OFs causes a significantly lower reduction in dissolved oxygen, compared to flooded field cultivation, due to the higher overall oxygen availability caused by SRI practices [125]. Immature mosquitoes do not rely on dissolved oxygen for respiration, and instead, sequester oxygen from the air through their spiracles [106]. Nevertheless, dissolved oxygen concentrations are important to larvae in general [126] but the exact impact on *Anopheles* species is uncertain.

A study in Ethiopia found increased levels of dissolved oxygen are positively associated with *Anopheles* larval abundances, but did not specify the species recorded [127]. Another study conducted along the Mara River found a strong positive correlation between dissolved oxygen concentrations and *Anopheles* larval abundance but again failed to identify the exact species present [89]. However, both corroborate with an earlier Kenyan study which demonstrated the same in *An. arabiensis* [128]*.* Similar results have been observed for *An. oswaldoi* in Venezuela [129] and *An. campestris* in Thailand [130]. These studies indicate dissolved oxygen plays an important role in *Anopheles* mosquito larval ecology, however, the species-specific effects have yet to be fully understood and whether these effects occur directly or indirectly, through modifying nutritional resources, such as algae and bacteria.

From these studies, it remains unclear how, when combined in SRI rice cultivation, the various modifiers of water physicochemistry may interact and impact the aquatic habitat’s biotic assemblage, including *Anopheles* mosquitoes. However, individually these practices have significant yet often conflicting effects on them.

#### Physical disturbance

In conventional rice cultivation, flooding is the primary means of weed control, however, the use of AWD in SRI necessitates manual weeding achieved through the use of a mechanical weeding tool or simply by hand [9]. Weeding frequency varies, but is most important in early growth when the younger rice plants are more susceptible to competitors [74]. As weed control in this manner is not typical of conventional cultivation, it is important to consider its potential impacts on mosquito ecology. Areas of consideration which require empirical investigation include the possible effects on eggs buried during weeding, whether the disturbance impacts their successful hatching, whether larvae or pupae can survive the physical action of a weeding tool or field-worker passing through their habitat, their responses to increased soil matter suspension, and how this might impact adult oviposition behaviour.

Little to no research has been dedicated to understanding how mechanical or manual weed removal may impact mosquitoes, though some has been conducted on how physical disturbance affects *Anopheles* mosquitoes. Although eggs of *Anopheles* mosquitoes have historically been assumed to hatch immediately after embryogenesis, physical agitation is required [131]. Considering that eggs laid by *Anopheles* mosquitoes in SRI rice fields would be exposed to wind and precipitation, the impact of weeding, no matter how frequent, would be unlikely to cause a dramatic increase in egg hatching, but in theory, it may have an impact, nonetheless. A study in the Philippines found *Anopheles* larval abundances were greatest in recently ploughed fields, and speculated it was due to reduced predation pressure [82], though agitation may play a role. A similar effect may be observed from the weed removal used in SRI cultivation.

## Conclusions

The cultivation of rice is well known to create favourable habitats for the breeding of *Anopheles* mosquitoes and is implicated in increased malaria incidences in associated communities. However, differences in cultivation practices and socioeconomic factors make this relationship complex. With the increasing pressures of a growing population, demand for rice, and climate change-induced water scarcity, SRI is promoted to simultaneously address these issues. Modifications to conventional rice cultivation, such as controlled irrigation, can modify the ecology of *Anopheles* mosquitoes and subsequently the transmission of malaria.

SRI modifies the local environment and, therefore, the mosquito habitat in ways not presently understood. If SRI continues to be promoted where malaria is endemic, we must seek to understand how the biotic and abiotic factors associated with *Anopheles* mosquitoes are modified, and how this may impact their ecology and biology. As these remain unknown, the potential for increasing mosquito productivity, inducing species composition changes, and the potential impact upon malaria transmission also remains unknown. The impacts of SRI on *Anopheles* mosquitoes have yet to be investigated and, therefore, research needs to be conducted to characterise the SRI rice cultivation system in terms relevant to the ecology of *Anopheles* mosquitoes. Foremost, field-based empirical research comparing SRI to conventional systems, in terms of *Anopheles* proliferation, must be conducted with urgency to inform the relative malaria risk SRI may pose, in the wake of its increasing adoption.

## Data Availability

Data supporting the main conclusions of this study are included in the manuscript.
